# Free triiodothyronine levels and age influences the metabolic profile and COVID-19 severity parameters in euthyroid and levothyroxine-treated patients

**DOI:** 10.3389/fendo.2022.1025032

**Published:** 2022-11-09

**Authors:** Inés Amich, Eduardo Anguita, Silvia Escribano-Serrat, Cristina Alvarez, Diego Rodríguez-Muñoz, Verónica García, Rocío Bello, José Alberto Peña-Pedrosa, Neus Martínez-Micaelo, Nuria Amigó, Pablo Ortiz, María José Torrejón, Lisardo Boscá, Javier Martín-Sánchez, Ana Aranda, Susana Alemany

**Affiliations:** ^1^ Department of Emergency, Hospital Príncipe de Asturias, Alcalá de Henares, Madrid, Spain; ^2^ Department of Hematology, Hospital Clínico San Carlos, Instituto de Medicina de Laboratorio (IML), Instituto de Investigación Sanitaria San Carlos (IdISSC), Madrid, Spain; ^3^ Department of Medicine, Universidad Complutense de Madrid (UCM), Madrid, Spain; ^4^ Clinical Analysis Laboratory, IML, IdISSC, Hospital Clínico San Carlos, Madrid, Spain; ^5^ Department of Metabolism and Cell Signaling, Instituto de Investigaciones Biomédicas “Alberto Sols”, CSIC-UAM, Madrid, Spain; ^6^ Hospital Pharmacy, IdISSC, Hospital Clínico San Carlos, Madrid, Spain; ^7^ Biosfer Teslab, Department of Basic Medical Sciences, Institut d'Investigació Sanitària Pere Virgili (IISPV), Reus, Spain; ^8^ Centro de Investigación Biomédicas en Red de Diabetes y Enfermedades Metabólicas Asociadas (CIBERDEM), Instituto de Salud Carlos III (ISCIII), Madrid, Spain; ^9^ Centro de Investigación Biomédicas en enfermedades Cardiovasculares (CIBERCV), Instituto de Salud Carlos III (ISCIII), Madrid, Spain; ^10^ Department of Emergency, IdISSC, Hospital Clínico San Carlos, Madrid, Spain; ^11^ Department of Endocrine and Nervous System Pathophysiology, Instituto de Investigaciones Biomédicas “Alberto Sols”, CSIC-UAM, Centro de Investigación Biomédicas en Red de Cáncer (CIBERONC), Madrid, Spain; ^12^ Centro de Investigación Biomédicas de Cáncer (CIBERONC), Instituto de Salud Carlos III (ISCIII), Madrid, Spain

**Keywords:** thyroid hormones, levothyroxine, age, 3-Hydroxybutyrate, cytokines, COVID-19 age, metabolism and COVID-19

## Abstract

Metabolic reprogramming is required to fight infections and thyroid hormones are key regulators of metabolism. We have analyzed in hospitalized COVID-19 patients: 40 euthyroid and 39 levothyroxine (LT4)-treated patients in the ward and 29 euthyroid and 9 LT4-treated patients in the intensive care unit (ICU), the baseline characteristics, laboratory data, thyroid-stimulating hormone (TSH), free thyroxine (FT4), free triiodothyronine (FT3), the FT3/FT4 ratio, 11 antiviral cytokines and 74 metabolomic parameters. No evidence for significant differences between euthyroid and LT4-treated patients were found in the biochemical, metabolomic and cytokines parameters analyzed. Only TSH (p=0.009) and ferritin (p=0.031) showed significant differences between euthyroid and LT4-treated patients in the ward, and TSH (p=0.044) and FT4 (p=0.012) in the ICU. Accordingly, severity and mortality were similar in euthyroid and LT4-treated patients. On the other hand, FT3 was negatively related to age (p=0.012), independently of sex and body mass index in hospitalized COVID-19 patients. Patients with low FT3 and older age showed a worse prognosis and higher levels of the COVID-19 severity markers IL-6 and IL-10 than patients with high FT3. IL-6 negatively correlated with FT3 (p=0.023) independently of age, body mass index and sex, whereas IL-10 positively associated with age (p=0.035) independently of FT3, body mass index and sex. A metabolomic cluster of 6 parameters defined low FT3 ward patients. Two parameters, esterified cholesterol (p=4.1x10^-4^) and small HDL particles (p=6.0x10^-5^) correlated with FT3 independently of age, body mass index and sex, whereas 3-hydroxybutyrate (p=0.010), acetone (p=0.076), creatinine (p=0.017) and high-density-lipoprotein (HDL) diameter (p=8.3x10^-3^) were associated to FT3 and also to age, with p-values of 0.030, 0.026, 0.017 and 8.3x10^-3^, respectively. In conclusion, no significant differences in FT3, cytokines, and metabolomic profile, or in severity and outcome of COVID-19, were found during hospitalization between euthyroid patients and hypothyroid patients treated with LT4. In addition, FT3 and age negatively correlate in COVID-19 patients and parameters that predict poor prognosis were associated with low FT3, and/or with age. A metabolomic cluster indicative of a high ketogenic profile defines non-critical hospitalized patients with low FT3 levels.

## Introduction

The coronavirus disease (COVID-19) caused by the Severe Acute Respiratory Syndrome type 2 (SARS-CoV-2) was first described in December 2019 in Wuhan, China, and rapidly spread worldwide (1, 2). In severe cases, SARS-CoV-2 causes bilateral pneumonia that progresses to acute respiratory distress syndrome and eventually death (2). Male sex and age, including its underlying comorbidities such as hypertension, diabetes mellitus, vascular, kidney and respiratory diseases, immunosuppressive conditions and cancer, have been described as bad prognostic factors (2, 3). Neutrophil-to-lymphocyte ratio, lactate dehydrogenase (LDH), D-dimer, C-reactive protein, creatinine, hemoglobin, aspartate aminotransferase (ALT), alanine aminotransferase (AST), ferritin, IL-6 and IL-10 among other parameters, indicate the severity of COVID-19 (4–6).

Hypothyroidism can be successfully overcome by Levothyroxine (LT4) sustitutive treatment leading in most cases to normal free triiodothyronine (FT3) levels (7). FT3 is a major endocrine regulator of the metabolic rate, increasing resting energy expenditure by directly regulating different metabolic pathways (reviewed in (8, 9). FT3 tends to be lower with age, a decrease believed to be beneficial in healthy individuals during old age by slowing-down metabolism (10, 11). Severely ill patients can also present a decrease in circulating T3, a condition denominated “non-thyroidal illness syndrome” or “euthryoid low T3 syndrome” (12, 13). Euthryoid low T3 syndrome has been reported in COVID-19 patients, although there is currently some discrepancy on the magnitude of the percentage of COVID-19 patients with low FT3 levels, ranging from 0% to 28% (14–21). Besides, low FT3 levels have been also associated with a poor prognosis in COVID-19 patients (19, 20).

In this study we have analyzed the association of FT3 levels and age, with biochemical data, metabolic parameters and cytokine levels involved in COVID-19 severity both in euthyroid patients and in hypothyroid patients on LT4 replacement therapy. We found a negative correlation between age and FT3 levels, and determined that increased COVID-19 severity markers are related either to higher age, low FT3 levels, or to both. We also define a specific metabolomic cluster, characteristic of a high ketogenic profile, in non-critical hospitalized COVID-19 patients with low FT3.

## Methods

### Study design and participants

This is a retrospective study and was approved by the Institutional Ethics Committee of the Hospital Clínico San Carlos (HCSC) (n°=20/397-E_COVID). The Biobank of the HCSC provided the serum samples and clinical data from COVID-19 patients (March 3^rd^ 2020-October 30^th^ 2020). First, we selected consecutive serum samples from LT4-treated COVID-19 patients with doses ≥ 525 mg/week in the ICU (n=9) and in the ward (n=39). Thirty-three % of these patients were athyreotic and the mean LT4 dose was 8.8 ± 2.7 mg/kg/week. Subsequently, samples from euthyroid COVID-19 patients in the ward (n=40) or in the ICU (n=29), matching the date of sampling (± 3-days), sex and age with their corresponding LT4-treated group, were chosen. Pregnant patients, patients with hyperthyroidism, with thyroid disorders not confirmed during hospitalization, with LT4 dose <525 mg/week and patients aged <18 years, were excluded from the study. 16.2% of the analyzed patients died in the hospital from COVID-19, either because of respiratory failure and lack of response to treatment, or because of secondary complications of the required invasive measures. Due to heparin prophylaxis, 2 patients died of hypovolemic shock secondary to rectorrhagia and 1 patient died from thrombosis with diffuse non-revascularized myocardial ischemia.

Additionally, severity parameters and outcome of 1,450 consecutive hospitalized patients registered in the COVID-19_URG-HCSC database were analyzed. The exclusion criteria, which were identical to those used for the choice of serum from COVID-19 patients, are shown in **Supplementary Figure 1**.

### Clinical laboratory tests

COVID-19 was confirmed by RT-PCR of SARS-CoV-2 RNA in nasopharyngeal and oropharyngeal samples performed in a QuantStudio-5 (Applied Biosystems^®^). Hemograms were performed on a DXH 900^®^ and biochemical parameters on an AU5800^®^ (Beckman-Coulter^®^). The coefficient of variation for the different parameters are shown in **Supplementary Table 1**. The cytokines included in the Human Anti-Virus Response Panel (740390, Biolegend^®^) were determined in serum samples according to the manufacturer´s instructions. Cytokines concentration was calculated using the Legendplex V8.0 software supplied by Biolegend^®^. TSH, FT4 and FT3 were assessed by chemiluminescence on a DXI-800^®^ (Beckman-Coulter^®^). A standard curve for each hormone or cytokine was performed in parallel with the analysis of the samples.

### ^1^H-NMR spectrophotometry analysis

For the metabolomic analysis by nuclear magnetic resonance spectroscopy (^1^H-NMR), frozen serum samples (250 μL) were shipped in dry ice to Biosfer Teslab^®^. ^1^H-NMR-spectra were recorded on a BrukerAvance III 600 spectrometer, with the following certifications and validations: PCT/EP2014/075873, ISO 9001:2015; IVD-CE, ISO 13485,2015; ISO 13485:2016; Spanish Medicines Agency and Health Products License number 6855-PS, 2016). The quality assurance systems are performed annually by the SGS company, certificate ES19/86886, 2019), and by TÜV Rheinland company, certificate 0.04.15155,2015).

Thirty-two lipoprotein parameters were determined. The analysis of lipoprotein profile was performed by the NMR-based Liposcale^®^ test. The lipid concentrations (i.e. triglycerides and cholesterol), size and particle number of the four main classes of lipoproteins [high-density lipoprotein (HDL), intermediate-density lipoprotein (IDL), low-density lipoprotein (LDL) and very-low-density lipoprotein (VLDL)], as well as the particle number of nine subclasses (large, medium and small VLDL, LDL, or HDL) were determined as previously reported (22, 23). Briefly, particle concentration and diffusion coefficients were obtained from the measured amplitudes and attenuation of their spectroscopically distinct lipid methyl group NMR signals, using the 2D diffusion-ordered ^1^H NMR spectroscopy (DSTE) pulse. The methyl signal was surface fitted with 9 lorentzian functions associated with each lipoprotein subclasses: large, medium and small of the main lipoprotein classes. The area of each lorentzian function was related to the lipid concentration of each lipoprotein subclass, and the size was calculated from their diffusion coefficient. Each subclass particle concentration was calculated by dividing the lipid volume by the particle volume of a given class. Lipid volumes were determined by using common conversion factors to convert concentration units into volume units (22, 23). The different lipoprotein subclasses correspond to the following diameter size ranges: large VLDL, 68.5 to 95.9 nm; medium VLDL, 47 to 68.5 nm; small VLDL, 32.5 to 47 nm; large LDL, 24 to 32.5 nm; medium LDL, 20.5 to 24 nm; small LDL, 17.5 to 20.5 nm; large HDL, 10.5 to 13.5 nm; medium HDL, 8.5 to 10.5 nm; and small HDL, 7.5 to 8.5 nm. Finally, weighted average VLDL, LDL and HDL particle sizes were calculated from various subclass concentrations by summing the known diameter of each subclass multiplied by its relative percentage of subclass particle number.

We also analyzed the region of the ^1^H-NMR spectrum where the glycoproteins resonate (2.15-1.90 ppm) using several analytical functions according to a previously published procedure (24). For each function, we determined the total area (proportional to concentration), height, position and bandwidth. The area of GlycA provided the concentration of acetyl groups of protein-bond N-acetylglucosamine and N-acetylgalactosamine, and the area of GlycB those of N- acetylneuraminic acid. GlycF area arises from the concentration of the acetyl groups of N-acetylglucosamine, N-acetylgalactosamine and N-acetylneuraminic acid unbound to proteins (free fraction). H/W ratios of GlycA and GlycB were also reported, being a parameter associated to the aggregation state of the sugar-protein bonds. Height was calculated as the difference from baseline to maximum of the corresponding NMR peaks and the width value corresponds to the peak width at half height. In total 6 glycoprotein parameters were determined.

A target set of 18 low molecular weight metabolites (LMWMs), including: isoleucine, leucine, valine, lactate, alanine, acetate, acetone, pyruvate, glutamine, creatine, creatinine, choline, proline, methanol, glycine, glycerol, serine, glucose, tyrosine, T-methylhistidine and formate, were identified and quantified in the 1D Carr-Purcell-Meiboom-Gill (CPMG) spectra using an adaptation of Dolphin. Each metabolite was identified by checking for all its resonances along the spectra, and then quantified using line–shape fitting methods on one of its signals (25, 26).

Lipophilic extracts were obtained from 200 μL aliquots of freshly thawed plasma using the BUME method (27) with slight modifications. BUME was optimized for batch extractions with di-isopropyl ether (DIPE) replacing heptane as the organic solvent, since the ^1^H-NMR fingerprint of heptane highly overlaps fatty acid signals. This procedure was performed with a BRAVO liquid handling robot which has capacity to extract 96 samples at once. The upper lipophilic phase was completely dried in Speedvac until evaporation of organic solvents and frozen at -80°C until NMR analysis. Lipid extracts were reconstituted in a solution of CDCl3:CD3OD: D2O (16:7:1, v/v/v) containing Tetramethylsilane (TMS) at 1.18 mM as a chemical shift reference and transferred into 5-mm NMR glass tubes. ^1^H-NMR spectra were measured at 600.20 MHz. A 90° pulse with water pre-saturation sequence (ZGPR) was used. Quantification of lipid signals in ^1^H-NMR spectra was carried out with LipSpin (28) an in-house software based on Matlab. Resonance assignments were done on the basis of literature values (29). The 18 lipid species obtained by this NMR approach included: cholesterol (free and esterified), unsaturated fatty acids (omega-6, omega-7, omega-9, omega-3), saturated fatty acids, monounsaturated fatty acids, linoleic acid, docosahexaenoic acid, arachidonic and eicosapentaenoic, glycerides and phospholipids (total cholines, triglycerides, phosphoglycerides, phosphatidylcholine, sphingomyeline and lysophosphatidylcholine).

### Identification of relevant metabolites and model building

A three-step multivariate analysis was applied to the ^1^H-NMR data to identify important metabolites and patterns for distinguishing between groups. In the first step, we applied 4 statistical approaches to identify the variables that make the largest contributions to the discrimination between groups. These approaches include the Wilcoxon rank-sum test correcting for multiple comparisons with the Benjamini-Hochberg procedure, the Spearman’s rank correlation, the Random Forest, and the Partial Least Squares discriminant analysis (PLS-DA). Those variables that resulted significant with a corrected p-value <0.05 were selected as the candidates for the Wilcoxon rank-sum test and the Spearman’s rank correlation. The 10 most important variables were determined by the variable importance score or the variable importance in projection (VIP) score using the Random Forest or the PLSA-DA, respectively. To avoid overfitting, a 10-fold cross-validation was performed. In the second step, using a Venn diagram we selected the most prominent metabolites included in the model, by determining those that overlap in at least three of the four statistical approaches. In the third step, we used a Principal Component Analysis (PCA) as an unsupervised method to visualize the capacity of the selected metabolites to separate the groups. Ellipses in PCA represent 95% confidence intervals around the centroid of each data cluster. Finally, we built a linear fitting model, by computing the area under the curve (AUC) and the 95% confidence interval of a receiver operating characteristics (ROC) curve. We evaluated and quantified how accurately the selected ^1^H-NMR variables were able to discriminate between groups. Patients were randomly assigned to training (70%) and test (30%) sets. We performed a 10-fold cross-validation with 100 replicates on the training data during the model construction process and tested the model on the hold-out data. Analysis was performed using the R statistical software version 4.1.1.

### Quantification and statistical analysis

Data were subjected to normality and lognormality tests. Two groups of unpaired variables with parametric distribution were compared using two-tailed Student’s *t* test and non-parametric distribution with two-tailed Mann-Whitney test. One-way ANOVA followed by multiple comparisons with Bonferroni test was used for multiple comparisons with unpaired parametric variables and with non-parametric distribution with the Kruskal-Wallis test followed by false discovery rate (FDR) by two-stage linear step-up procedure of Benjamini, Krieger and Yekutieli tests. Parametric data are presented as means ± S.D and non-parametric as medians and interquartile range 1-3 [Q1;Q3]. The X^2^ or two-sided Fisher’s exact tests were used to evaluate the differences of categorical variables, presented as percentages. Multivariate linear regression models were used to analyze the independent association of FT3 with age and ICU, and with some cytokines and metabolomic parameters, adjusting for sex and body mass index (BMI). Statistics were performed with GraphPad Prism 7.0 or R statistical software. Differences between the groups were considered significant when: *p<0.05, **p<0.01, or ***p<0.001.

## Results

### Thyroid hormones, cytokines and metabolomic profile in euthyroid and LT4-treated patients during COVID-19 hospitalization

TSH, FT4 and FT3 levels were determined in euthyroid and LT4-treated ward hospitalized COVID-19 patients and in euthyroid and LT4-treated ICU COVID-19 patients. **Table 1** shows the baseline characteristics for these 4 groups. The ratio of females to males did not reflect evidence for significant differences among groups. Furthermore, no significant differences in age were observed between the two ward groups and between the two ICU groups, although both groups of ward patients were significantly older than those in the ICU (euthyroid p=0.006, LT4-treated p=0.004). Among the comorbidities analyzed, hypertension tended to be higher in ward LT4-treated patients and chronic renal disease was higher in all LT4-treated patients. Biochemical laboratory data obtained on the day of blood sampling for hormones determination, revealed that the only significant difference detected between the two ward groups was a lower ferritin value in the LT4-treated group (p=0.031). No evidence for statistical differences was found between the two ICU groups. The comparison between ward and ICU groups reflected a more severe biochemical COVID-19 profile in the ICU patients. The euthyroid ward patients presented a lower count of leukocytes (p=0.0008), neutrophils (p=0.001), neutrophil-to-lymphocyte ratio (p=0.004) and platelets (p=0.018) than the euthyroid ICU group. The euthyroid ward group also displayed lower levels of ALT (p=0.006), C-reactive protein (p=0.015) and fibrinogen (p=0.012) than the euthyroid ICU group (**Table 1**). Fewer statistical differences between LT4-treated ward and ICU groups were found, most likely due to the low number of patients in the LT4-treated ICU group. The percentage of patients taking medication that might affect thyroid function (30, 31) on the day before blood sampling for hormones was high and with no evidence for significant differences between groups (**Supplementary Table 2**).

**Table 1:** Baseline characteristic and laboratory data in hospitalized COVID-19 patients.

	Ward	Ward	ICU	ICU	
	Euthyroid (n = 40)	LT4-treated (n = 39)	Euthyroid (n = 29)	LT4-treated (n = 9)	p-value
*Socio-demographic data and medical history*					
Male (n=16,17,16, 5)	40%	43.60%	57.10%	55.50%	0.576
Female (n=24,23,13,4)	60%	56.40%	42.80%	38.50%	0.519
Age, years (n=40,39,29,9)	69 ± 4.8	70.7 ± 13.8	60.9 ± 11.6**	56.1 ± 11.3##	0.001
BMI, kg/m^2^ (n=25,30,25, 6)	28.4 ± 4.6	29.3 ± 5.1	31.9 ± 6.1	34.0 ± 10	0.136
Tobacco (n=4,5,1,1)	10.0%	12.8%	3.4%	11.1%	–
Diabetes (n=5,12,8,1)	16.5%	30.8%	27.6%	11.1%	0.174
Hypertension (n=13,23,9,2)	32.5%	59.8%	31.0%	22.2%	0.050
Cancer (n=6,8,1,0)	15.0%	20.5%	3.4%	0.0%	0.119
CODP (n=4,4,1,1)	10.0%	10.3%	3.4%	11.1%	–
Chronic renal disease (n=3,8,2,3)	7.5%	30.8%	6.9%	33.3%	0.074
Cardiopathy (n=9,10,3,0)	22.5%	25.6%	10.3%	0.0%	0.172
Cerebrovascular disease (n=1,1,0,0)	2.5%	2.5%	0.0%	0.0%	–
*Laboratory data at the day of TSH, FT4 and FT3 and TH analysis*					
Leukocytes × 10^3^/µL (n=40,39,29,9)	5.8 [4.1;9.2]	7.1 [4.8;10.3]	8.5 [7.4;11.6] ***	10.2 [6.8;11.6]	0.002
Hemoglobin g/dL (n=40,39,29,9)	12.0 [10.1;13.3]	12.3 [10.4;13.4]	11.2 [9.8;13.1]	10.3 [8.8;12.8]	0.206
Lymphocytes × 10^3^/µL(n=40,39,29,9)	0.6 [0.3;1.1]	0.75 [0.4;1.1]	0.5 [0.2;0.9]	0.5 [0.2;0.9]	0.379
Neutrophils × 10^3^/µL (n=40,39,29,9)	3.7 [2.3;6.8]	5.1 [2.8;7.6]	6.8 [4.2;9.1]**	8.9 [5.1;9.8]*#*	0.002
Neutrophils/Lymphocytes ratio	3.3 [1.7;6.2]	3.8 [1.8;8.2]	7.2 [3.5;15.3]**	10.7 [3.9;21.1]#	0.003
Platelet count × 10^3^/µL (n=40,39,29,9)	226.5 [164.8;336.0]	211 [147;303]	285 [233;383.8]*	121 [381;451]##	0.002
ALT U/L (n=40,39,29,9)	27.8[15.4;71.3]	22.2 [16.1;42.8	52.6 [28.5;97.1] **	50.9 [24.5;86.8]#	0.001
AST U/L (n=40,39,29,9)	27.5 [21.0;47.5]	26.1 [18.5;39.5]	37.4 [22.6;61.9]	40.8 [23.5;57.6]	0.030
C-reactive protein mg/dL (n=40,39,29,9)	2.6 [0.8;8.2]	2.7 [0.7;9.1]	7.4 [3.0;13.5]*	1.8 [0.7;15.5]	0.065
D-Dimer ng/dL (n=40,39,29,9)	928 [705;4,875]	1,238 [526;1,328]	1,342 [784;2,245]	2,294[1,294;5,318]#	0.164
Fibrinogen mg/dL (n=40,39,29,9)	583 [462;662]	505 [360;681]	728.5 [590.8;846]*	510 [429.5;829.5]	<0.0001
Ferritin ng/dL (n=40,39,29,9)	612 [312;1003]	292 [121;587] $	654 [436;1,133]	588 [233;841]	0.001
LDH U/L (n=40,39,29,9)	556 [439;730]	503 [421;641]	691 [522;899] =0.07	821 [557;1142]##	0.001

Non-categorical data are shown as percentages and analyzed by X^2^. Parametric age and BMI are shown as means+S.D and were analyzed by one-way ANOVA followed by multiple comparisons with Bonferroni test Non-parametric data are expressed as medians and interquartile range [Q1;Q3] and comparisons were made with Kruskal-Wallis test followed by false discovery rate by two-stage linear step-up procedure of Benjamini, Krieger and Yekutieli to compare the different groups two by two. No statistical differences between euthyroid ICU and LT4-treated ICU patients were found. In the table p-values correspond to the ANOVA analysis and multiple comparisons are indicated as: $p < 0.05 between euthyroid ward and LT4-treated ward patients; *p < 0.05, **p < 0.01 and ***p < 0.001 between euthyroid ward and euthyroid ICU patients; #p < 0.05, ##p < 0.01 between LT4-treated ward and LT4-treated ICU patients. -: Low number for X^2^ calculation; AST, Aspartate aminotransferase; ALT, Alanine aminotransferase; BMI, Body mass index; COPD, chronic obstructive pulmonary disease; LDH, Lactate dehydrogenase.

**Figure 1:**
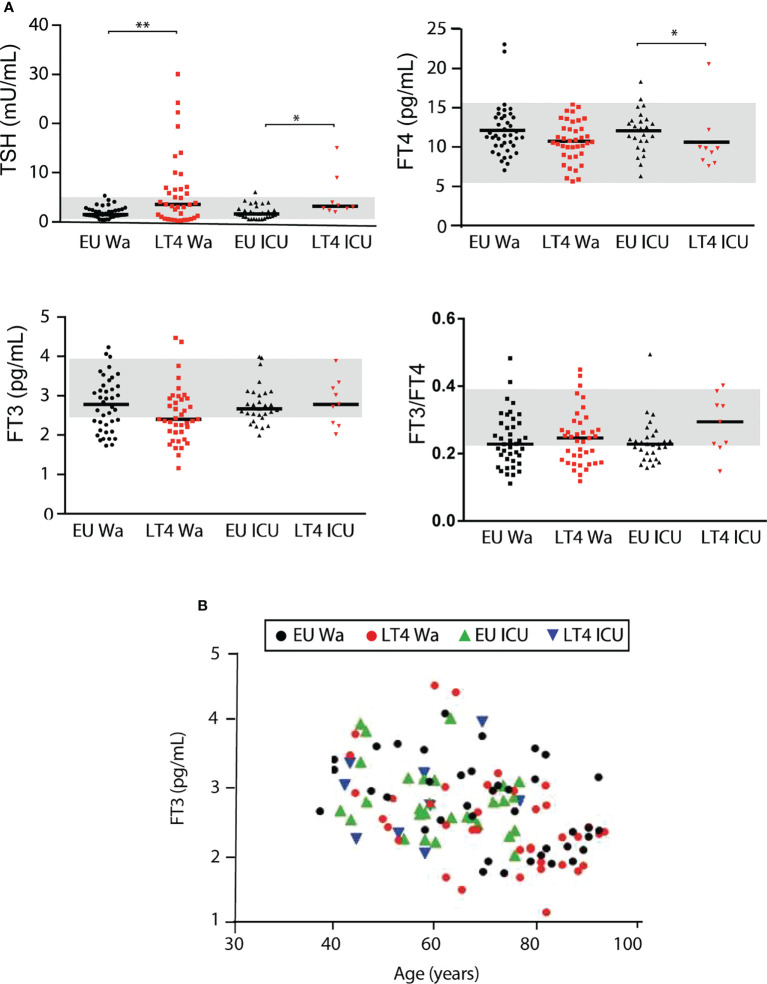
TSH, FT4 and FT3 levels in euthyroid and LT4-treated patients during COVID-19 hospitalization. **(A)** TSH, FT4, FT3 and FT3/FT4 in euthyroid and LT4-treated (> 525 mg/week) COVID-19 hospitalized patients. Comparisons were made with Kruskal-Wallis test followed by FDR by two-stage linear step-up procedure of Benjamini, Krieger and Yekutieli. Only statistical differences between both ward or ICU groups, or between the two euthyroid or LT4-treated groups were considered. Grey bands represent physiological hormone range. ∗p < 0.05, ∗∗p < 0.001. **(B)** Correlation FT3 versus age in the groups indicated in A.

TSH levels were higher in LT4-treated groups than in euthyroid groups, either in the ward (p=0.009) or in the ICU (p=0.044). We found no evidence for significant differences in FT4 and FT3 levels or in the FT3/FT4 ratio between euthyroid and LT4-treated patients in the ward, but LT4-treated ICU patients displayed lower FT4 than their euthyroid counterparts (p=0.014) (**Figure 1A**; **Supplementary Table 3**). FT3 decreased with age in COVID-19 patients. Multivariate linear regression analysis showed that FT3 remained a significant variable related to age (p=0.012), independently of being in the ward or ICU (interaction p= 0.198), as well as of sex and BMI (**Figure 1B**; **Table 2**).

**Table 2:** Multivariate linear regression analysis of FT3 with age, ICU and their interaction-term, adjusted for sex and BMI in hospitalized COVID-19 patients.

	Beta	95% CI*^1^*	p-value
Sex (m *vs* f)	0.016	-0.243, 0.275	0.902
BMI (kg/m^2^)	0.005	-0.018, 0.028	0.681
Age (y)	-0.016	-0.028, -0.004	0.012
ICU (yes *vs* no)	-0.901	-2.402, 0.600	0.236
Age (y) ICU vs. Ward	0.015	-0.008, 0.038	0.198

BMI, Body mass index; CI^1^, Confidence Interval; ICU, Intensive care unit; Vs, Versus.

Analysis of 11 antiviral-related serum cytokines indicated no evidence for significant differences between euthyroid and LT4-treated patients either in the ward or in the ICU. However, euthyroid and LT4-treated patients in the ward displayed significantly higher levels of IL-1β and lower levels of IP-10, IFN-β, IFN-γ and GM-CSF than the corresponding ICU groups. IL-8 and IL-12 p70 were only significantly higher in the euthyroid ward group, probably due to the low number of patients in the ICU LT4-treated group (**Table 3**).

**Table 3:** Circulating antiviral cytokine levels in hospitalized COVID-19 patients.

	Ward	Ward	ICU	ICU	
	Euthyroid (n = 40)	LT4-treated (n = 39)	Euthyroid (n = 29)	LT4-treated (n = 9)	p-value
IL-1β (pg/mL)	296 [144;08]	268 [106;490]	99 [48;121]***	152 [102;396]#	<0.0001
IL-6 (pg/mL)	76 [2.4;573]	2.4 [1.9;365]	138 [89;304]	102 [39;185]	0.074
IL-8 (pg/mL)	11 [2.8;20]	8.0 [1.2;23]	21 [15;24]**	14 [18.4;21]	0.003
IL-10 (pg/mL)	5.1 [14.0;5.8]	4.5 [4.0;5.4]	5.4 [3.8;18]	2.3 [0.1;8.4]	0.166
IL-12 p70 (pg/mL)	38 [1.9;109]	2.9 [1.9;75]	94 [48;121]**	52 [26;89]	0.001
TNF-α (pg/mL)	324 [77;1,555]	158 [78;548]	140 [64;320]	126 [5.4;492]	0.216
IP-10 (pg/mL)	0.7 [0.5;1.4]	0.7 [0.6;1.1]	43 [26;88]***	55 [39;96]###	< 0.0001
IFN-β (pg/mL)	1.6 [1.5;2.1]	1.7 [1.5;2.1]	61 [26;96]***	65 [34;89] ###	< 0.0001
IFN-γ (pg/mL)	3.0 [1.2;53]	1.9 [1.2;5.5]	15 [12;38]***	13 [7.6;32]##	< 0.0001
GM-CSF (pg/mL)	1.5 [1.3;1.9]	1.6 [1.37;1.9]	14 [5.6;23]***	2.8 [2.5;25]##	< 0.0001

Cytokine medians and interquartile range [Q1;Q3] are shown. Comparisons were made with Kruskal-Wallis test followed by false discovery rate by two-stage linear step-up procedure of Benjamini, Krieger and Yekutieli. Only statistical differences between both ward or ICU groups, or between the two euthyroid or LT4-treated groups were considered. No significant differences between euthyroid ward and LT4-treated ward patients and between euthyroid ICU and LT4-treated ICU patients were found. **p < 0.01, ***p < 0.001 between euthyroid ward and euthyroid ICU patients. #p < 0.05, ##p <0.01, ###p < 0.001 between LT4-treated ward and LT4-treated ICU patients.

**Figure 2:**
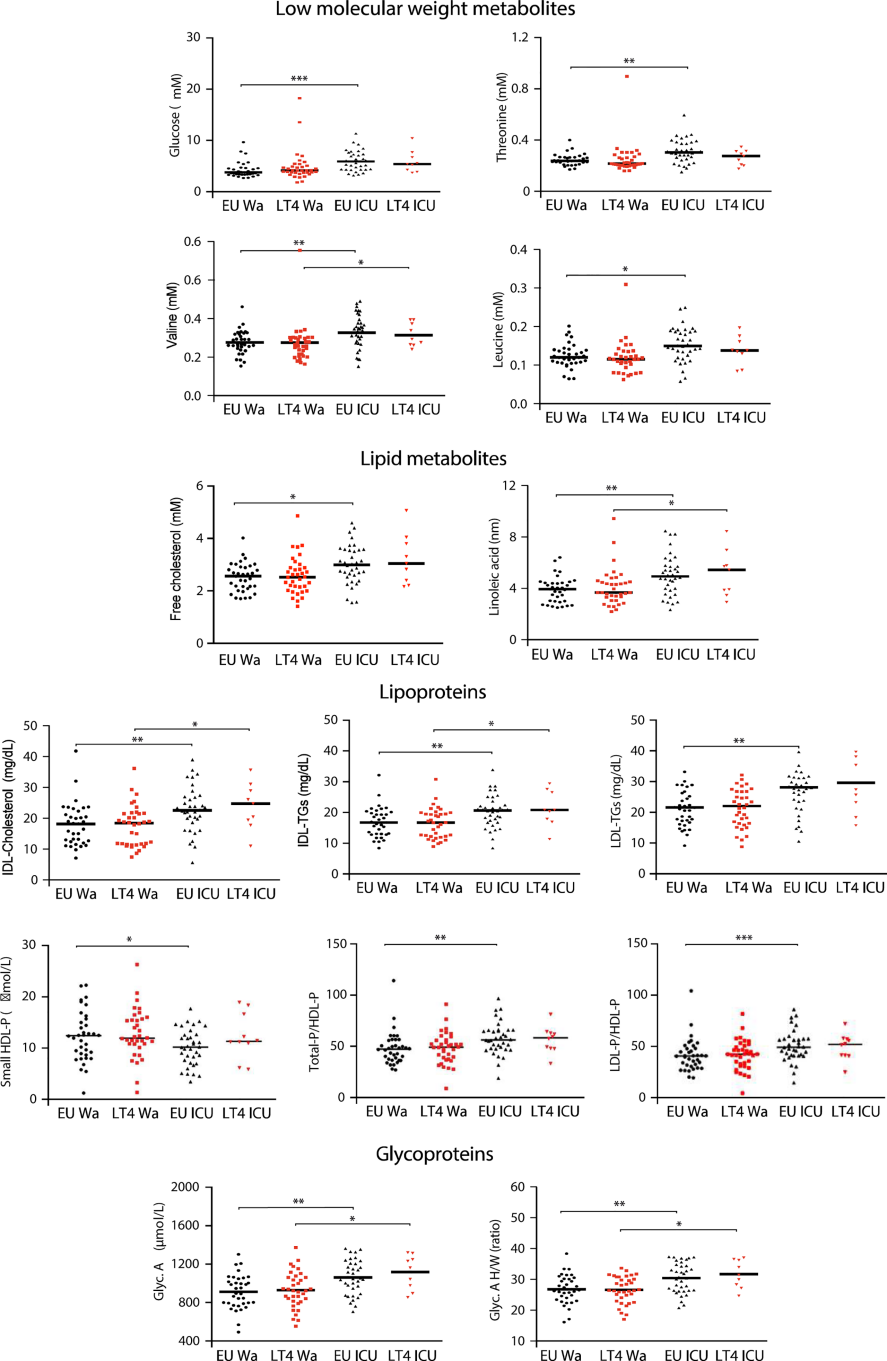
Serum metabolomic profile of euthyroid and LT4-treated hospitalized COVID-19 patients. Metabolomic parameters with statistically significant differences between groups found in **Supplementary Table 4**. Comparisons were made with Kruskal-Wallis test followed by FDR by two-stage linear step-up procedure of Benjamini, Krieger and Yekutieli. Only statistical differences between both ward or ICU groups, or between the two euthyroid or LT4-treated groups were considered. *p<0.05, **p < 0.01, ***p < 0.001. IDL, Intermediate density lipoprotein; TGs, Triglycerides; LDL, Low density lipoprotein; HDL-P, High density lipoprotein-particle; Glyc, Glycoprotein; H/W, High/width.

We detected 18 low molecular weight metabolites, 18 lipids, 32 lipoproteins and 6 glycoprotein parameters by ^1^H-NMR in serum from ward and ICU COVID-19 patients (**Supplementary Table 4**). There was no evidence for significant differences in the metabolomic profile between euthyroid and LT4-treated ward hospitalized patients or between the two ICU groups. The main differences were found between euthyroid ward and ICU groups and again statistical differences between both LT4-treated groups in some of these parameters were not obtained, likely due to the low number of LT4-treated ICU patients. The levels of the low molecular weight metabolites glucose, threonine, valine and leucine were significantly lower in the euthyroid ward group than in the euthyroid ICU group (p-values <0.0001, 0.001, 0.001 and 0.024, respectively). Free cholesterol and linoleic acid were the lipid metabolites that showed statistical differences between the euthyroid groups in the ward and in the ICU (p=0.025 and p=0.001, respectively). Concerning the lipoprotein profile, euthyroid ICU patients presented higher levels of intermediate density lipoprotein (IDL)-cholesterol (p=0.006) and IDL-triglycerides (TGs) (p=0.009) than euthyroid ward patients. Glycoprotein A and their high/width ratio were also increased in the euthyroid ICU group with respect to the ward group (p=0.0071, p=0.0028, respectively) (**Figure 2**; **Supplementary Table 4**).

All these data indicate no evidence for significant differences between patients treated with LT4 or not, in terms of the cytokine and metabolomic profiles tested. To further confirm the similarity between euthyroid patients and patients under LT4 treatment, we compared COVID-19 severity and outcome of 1,266 euthyroid patients and 85 LT4-treated patients out of a total of 1,450 patients with laboratory-confirmed COVID-19, admitted consecutively to the Hospital Emergency Department (**Supplementary Figure 1**). The higher prevalence of the thyroid disease in females reflected their higher percentage in the LT4-treated group (p=<0.0001), with no evidence for statistical differences in age. Related to the severity of COVID-19 during hospitalization, there was no evidence for significant differences in pneumonia consolidation and oxygen therapy requirements between both groups. More importantly, between euthyroid and LT4-treated patients there was no evidence for significant differences in 60-day and 30-day all-cause hospital mortality and in severity score, measured as ICU admission/in-hospital mortality/high oxygen requirement rate (**Table 4**).

**Table 4:** Gender, age, severity and mortality within 30 and 60 days in hospitalized COVID-19 patients.

	Admission	Admission	
	Euthyroid (n = 1,266)	LT4-treated (n = 85)	p-value
*Gender and age*			
Male (n=740,25)	58.5%	29.4%	<0.0001
Female (n=526,60)	41.5%	70.6%	<0.0001
Age (n=1166,85)	66.4+/-17.6	70.2+/-13.6	0.49
*Chest X-ray findings*			
No consolidation (n=237,25)	18.7%	21.2%	X^2 =^ 0.73
Unilateral consolidation (n=317,33)	25.0%	28.5%	
Bilateral consolidation (n=694,42)	54.8%	49.4%	
No performed (n=18,2)	1.4%	1.7%	
*Oxygen therapy*			
no oxygen therapy required (n=438,36)	34.7%	30.6%	X^2 =^ 0.58
Nasal cannula (n=404,40)	32.7%	34.1%	
Venturi mask (n=92,4)	7.3%	3.4%	
Reservoir (n=208,18)	16.5%	15.3%	
High flow (n=8,0)	0.6%	0.0%	
NIPPV (n=23,1)	2.6%	0.85%	
TPAP (n=2,0)	0.2%	0.0%	
Mechanical ventilation (n=57,7)	4.5%	6.0%	
No specified (n=19,4)	1.5%	3.4%	
*Severity*			
In-hospital mortality (n=216,18)	16.8%	15.3%	0.88
30-day mortality (n=222,18)	17.5%	15.3%	0.66
60-day mortality (n=243,20)	19.2%	17.0%	0.89
ICU/inside-hospital mortality/high			
rate of O_2_ requirement (n=308,22)	35.5%	35.5%	1

Non-categorical data are entered as percentages and were analyzed either by Fisher exact test (two groups) or by X^2^ (more than two groups). Parametrical data are shown as means ± S.D and were analyzed with two-tailed Student´s t test. NIPPV, Non-invasive Positive Pressure Ventilation; TPAP, Two-levels of Positive Airway Pressure.

### Influence of FT3 levels in the cytokine and metabolomic profiles in non-critical hospitalized COVID-19 patients

Low FT3 levels have been associated with a poor prognosis in COVID-19 (19, 20). To study the impact of FT3 levels on disease severity, as well as on the cytokine and metabolomic profiles during non-critical COVID-19 hospitalization, euthyroid and LT4-treated ward patients were clustered according to their circulating FT3 levels. The low-FT3 group (<2.1 pg/mL) consisted of 18 ward patients, 9 of them euthyroid; while the high-FT3 group (>3.0 pg/mL) comprised 17 ward patients, 12 of them euthyroid. No evidence for significant differences in TSH and FT4 values between both groups were found, while the statistical difference for FT3 levels or FT3/FT4 ratio was p=<0.0001 (**Supplementary Figure 2**). In agreement with the negative association between age and FT3 levels, the low-FT3 group was 15 years older on average (p=0.007) and presented a higher incidence of diabetes (p=0.045), hypertension (p=0.017) and cardiopathy (p=0.004), lower hemoglobin (p=0.046) and lymphocyte count (p=0.006), as well as a higher neutrophil-to-lymphocyte ratio (p=0.037) and C-reactive protein levels (p=0.013). Other severity parameters also tended to be more elevated in patients with low-FT3, including the percentage of *exitus*, which was 3 times higher, although the difference did not reach statistical significance, most likely due to the small number of patients (**Table 5**).

**Table 5:** Baseline characteristics, laboratory data and outcome in ward hospitalized COVID-19 patients.

	Low-FT3 (n = 18)	High-FT3 (n = 17)	p-value
*Gender, age and medical history*			
Male (n=7,9)	38.9%	52.9%	0.505
Female (n=11,8)	61.1%	47.1%	0.505
Age, years (n=18,17)	78.1 ± 7.7	63.6 ± 13.6	0.007
BMI, kg/m^2^ (n=13,12)	31.9 ± 16.6	30.1 ± 4.2	0.162
Tobacco (n=1,1)	5.6%	5.7%	>0.9999
Diabetes(n=5,0)	27.8%	0.0%	0.045
Hypertension (n=14,6)	77.8%	35.3%	0.017
Cancer (n=4,3)	23.5%	17.2%	>0.9999
CODP (n=1,0)	5.6%	0.0%	>0.9999
Chronic renal disease (n=3,8,2,3)	0.0%	23.5%	0.228
Cardiopathy (n=11,2)	61.1%	11.7%	0.004
Cerebrovascular disease (n=0,0)	0.0%	0.0%	>0.9999
*Laboratory data at the day of TSH, FT4 and FT3*			
Leukocytes × 10^3^/µL (n=18,17)	7.25 [4.3;10.9]	6.5 [5.6;10.0]	0.688
Hemoglobin g/dl (n=18,17)	10.8 [9.6;12.2]	12.2 [10.6;15.6]	0.046
Lymphocytes × 10^3^/µL (n=18,17))	0.8 [0.5;1.1]	1.3 [0.8;1.9]	0.006
Neutrophils × 10^3^/µL (n=18,17)	5.6 [2.8;9.15]	4.4 [2.9;14.7]	0.215
Neutrophils/Lymphocytes ratio	6.7 [3.5;18.10]	3.7 [2.0;5.5]	0.037
Platelet count × 10^3^/µL (n=18,17)	263.5 [181.5;431.3]	209 [152.0;280.0]	0.106
ALT U/L (n=18,17)	19.2 [12.0;38;9]	43.1 [14.5;75.2]	0.090
AST U/L (n=18,17)	28 [16;51]	23.5 [20.2;34]	0.941
C-reactive protein mg/dL (n=18,17)	9.6 [1.4;13.8]	1.34 [5.4;0.6]	0.013
D-Dimer ng/dL (n=18,17)	1,404 [545;4,564]	458 [281;5,268]	0.319
Fibrinogen mg/dL (n=18,17)	656 [499;714]	536 [396;633]	0.136
Ferritin ng/dL (n=18,17)	678.8 [269.3;1,098]	594.9 [163.4;763.5]	0.275
LDH U/L (n=17,18)	518 [418;955]	525 [457.3;597]	0.960
*Outcome*			
*Exitus* (n=6,2)	33.3%	11.7%	0.147

Non-categorical data are entered as percentages and were analyzed by Fisher exact test. Parametrical data are shown as means ± S.D and were analyzed by two-tailed Student´s t test. Non-parametric data are expressed as medians and interquartile range [Q1;Q3] and comparisons were made with Mann-Withney test. AST: Aspartate aminotransferase; AST, Aspartate aminotransferase; ALT, Alanine aminotransferase; BMI, Body mass index; COPD, chronic obstructive pulmonary disease; LDH, Lactate dehydrogenase.

The FT3/FT4 ratio has been found to be associated with poor prognosis in several diseases (32–34), including COVID-19 (17, 35). Thus, we also clustered the ward patients into high and low FT3/FT4 groups to compare COVID-19 severity. The. low FT3/FT4 group with a ratio <0.172 consisted of 18 patients, 9 of which were on LT4 treatment, and the high FT3/FT4 group with a ratio >0.303, was composed of 17 patients, 8 of which were on LT4 treatment. The low-FT3/FT4 group was significantly older (p<0.001) and had an elevated neutrophil-to-lymphocyte ratio (p<0.001), as well as higher levels of C-reactive protein (p=0.047) and D-dimer (p=0.015). The percentage of *exitus* in the low FT3/FT4 group was of 27.8% and in the high FT3/FT4 group was of 5.8% but, as in the case of the low-FT3 patients, this difference did not reach statistical significance. (**Supplementary Table 5**).

In the analysis of the antiviral cytokines, only two cytokines were significantly higher in the low-FT3 group, IL-6 (p=0.016) and IL-10 (p=0.015) (**Table 6**), both defined as markers of COVID-19 severity (6). IL-6 levels were also higher (<0.049) in the low FT3/FT4 group (**Supplementary Table 5**). Multilinear regression analysis revealed that only IL-6 was negatively associated with FT3 (p=0.023) independently of age, sex and BMI, whereas IL-10 was positively associated with age (p=0.035) independently of sex, BMI and FT3 (**Table 7**).

**Table 6:** Circulating antiviral cytokines levels in ward hospitalized Covid-19 patients.

	Low-FT3 (n = 18)	High-FT3 (n = 17)	p-value
IL1-β (pg/mL)	419[142;908]	285[106;676]	0.290
IL-6 (pg/mL)	450[2.4;2057]	2.5[1.8;253]	0.016
IL-8 (pg/mL)	9.5[4.8;34]	10[1.3;15]	0.507
IL-10 (pg/mL)	5.2[4.1;19]	4.5[4.0;4.9]	0.015
IL-12p70 (pg/mL)	85[2.3;191]	2.6[1.9;94]	0.054
TNF-α (pg/mL)	216[94;3563]	221[22;849]	0.315
IP-10 (pg/mL)	0.8[0.5;2.1]	0.7[0.6;1.2]	0.687
IFN-β (pg/mL)	1.8[1.5;29]	1.7[1.5;1.8]	0.251
IFN-γ (pg/mL)	3.4[2.1;97]	2.0[1.1;29]	0.290
GM-CSF (pg/mL)	1.8[1.3;2.5]	1.5[1.3;2.0]	0.558

Medians and interquartile range [Q1;Q3] of the indicated cytokines are shown. Comparisons were made with Mann-Withney test.

**Table 7:** Multivariate linear regression analysis of IL-6 and IL-10 according to FT3 with adjustment for age, sex, and BMI in ward hospitalized COVID-19 patients.

	Beta	95% CI^1^	p-value
*IL-6*			
Sex (m *vs* f)	0.12	-0.228, 0.469	0.669
BMI (kg/m^2^)	0.031	-0.005, 0.065	0.086
Age (y)	-0.012	-0.026, -6.9x10^-4^	0.062
FT3 (pg/mL)	-3.9x10^-4^	-7.7x10-4, -6.5x10^-5^	0.023
*IL-10*			
Sex (m *vs* f)	0.026	-0.326, 0.377	0.884
BMI (kg/m^2^)	0.021	-0.016, 0.058	0.255
Age (y)	-0.015	-0.029, -0.001	0.035
FT3 (pg/mL)	-2.7x10^-4^	-0.005, 0.006	0.920

Multivariate linear regression models were constructed to assess independent associations between IL-6 and IL-10 cytokine levels as variables and FT3 levels, with adjustment for age, sex, and BMI among ward hospitalized patients. BMI, Body mass index; CI^1^, Confidence Interval. Vs, versus.

**Figure 3:**
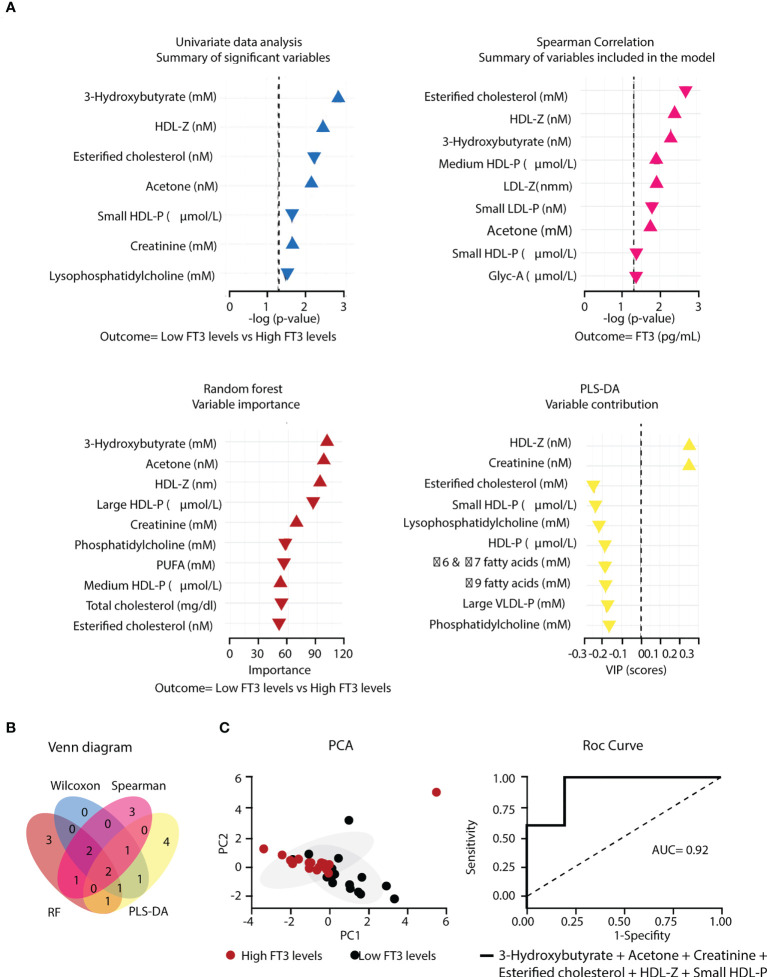
Metabolomic cluster associated to low FT3 in COVID-19 ward hospitalized patients. **(A)** Variables differently expressed in the low-FT3 group (n=18) and high-FT3 group (n=17) determined by four different methods. **(B)** Venn diagram describing the overlapping metabolomic parameters from the 4 statistical models. **(C)** PCA based on two principal components using the metabolomic cluster resulting from the previous steps, and Roc curve showing the accuracy (AUC). Gly, Glycoprotein; P, Particles; PUFA Polyunsaturated fatty acids (four PUFA signals); ω, Omega; Z, Diameter.

To identify whether variations in circulating FT3 levels also correlated with changes in the metabolomic pattern of non-critical hospitalized COVID-19 patients, a three-step data analysis was performed on the metabolomic parameters obtained from the low-FT3 and high-FT3 groups (**Supplementary Table 6**). In a first step, we applied 4 statistical approaches to identify the variables that make the largest contributions to the discrimination between groups (**Figure 3A**). In the second step, using a Venn diagram, we selected the metabolites that overlap in at least three of the four statistical approaches. Six variables: 3-hydroxybutyrate, acetone, creatinine, esterified cholesterol, HDL-Z, and small HDL-P were obtained (**Figure 3B**). In the third step, this cluster was further visualized by PCA, which presented different PC1 and PC2. The evaluation of the model performance by a ROC curve showed an AUC of 0.92 (95% CI = 0.74-1). In addition, the accuracy of the obtained model was 78.0% (accuracy>no information rate) p=0.157 and an out-of-bag error (OOB) of p=0.27. All these data indicate a robust association of the metabolomic cluster constituted by 3-hydroxybutyrate, acetone, creatinine, esterified cholesterol, and HDL-Z with the non-critical hospitalized low-FT3 group compared to the high-FT3 group (**Figure 3C**). Multivariate linear regression models with all the ward hospitalized patients were constructed to assess independent associations between FT3 and these metabolomic variables, after adjustment for age, sex and BMI. Using these models, FT3 was directly related to the levels of esterified cholesterol (p=4.1x10^-4^) and small HDL particles (6.0x10^-5^), while an inverse association was found for 3-hydroxybutyrate (p=0.010), creatinine (p=0.017), HDL-Z (p=3.6x10^-4^) and acetone (p=0.076). Age also remained a significant variable for creatinine (p=0.012), HDL-Z (p=8.3x10^-3^), acetone (p=0.026) and 3-hydroxybutyrate (p=0.030) in ward patients (**Table 8**).

**Table 8:** Multivariate linear regression analysis of metabolomic variables according to FT3, with adjustment for age, sex and BMI.

	Beta	95% CI^1^	p-value
*3- Hydroxybutyrate*			
Sex (m *vs* f)	0.208	-0.230, 0.645	0.34
BMI (kg/m^2^)	0.014	-0.035, 0.064	0.563
Age (y)	-0.023	-0.044, 0.002	0.030
FT3 (pg/mL)	-2.536	-4.434, -0.638	0.010
*Acetone*			
Sex (m *vs* f)	0.189	-0.245, 0.622	0.383
BMI (kg/m^2^)	0.009	-0.043, -0.061	0.732
Age (y)	-0.025	-0.047, -0.003	0.026
FT3 (pg/mL)	-4.700	-9.913, 0.514	0.076
*Creatinine*			
Sex (m *vs* f)	0.294	-0.128, 0.716	0.166
BMI (kg/m^2^)	-0.003	-0.050, 0.044	0.904
Age (y)	-0.027	-0.048, -0.006	0.012
FT3 (pg/mL)	-4.311	-7.811, -0.811	0.017
*Esterified cholesterol*			
Sex (m *vs* f)	0.123	-0.253, 0.499	0.511
BMI (kg/m^2^)	0.008	-0.035, 0.052	0.697
Age (y)	-0.015	-0.035, 0.006	0.152
FT3 (pg/mL)	0.412	0.198, 0.625	4.1x10^-4^
*HDL-Z*			
Sex (m *vs* f)	0.072	-0.305, 0.449	0.700
BMI (kg/m^2^)	-0.001	-0.044, 0.042	0.970
Age (y)	-0.026	-0.045, -0.007	8.3x10^-3^
FT3 (pg/mL)	-1.677	-2.536, 0.817	3.6x10^-4^
*small HDL-P*			
Sex (m *vs* f)	0.104	-0.253, 0.461	0.556
BMI (kg/m^2^)	0.022	-0.020, 0.065	0.297
Age (y)	-0.018	-0.037, -0.001	0.057
FT3 (pg/mL)	0.076	-0.042, 0.110	6.0x10^-5^

Multivariate linear regression models were constructed to assess independent associations between metabolomic variables and FT3, after adjustment for age, sex, and BMI in ward hospitalized COVID-19 patients. BMI, Body mass index; CI^1^, Confidence Interval; Vs, versus.

**Figure 4:**
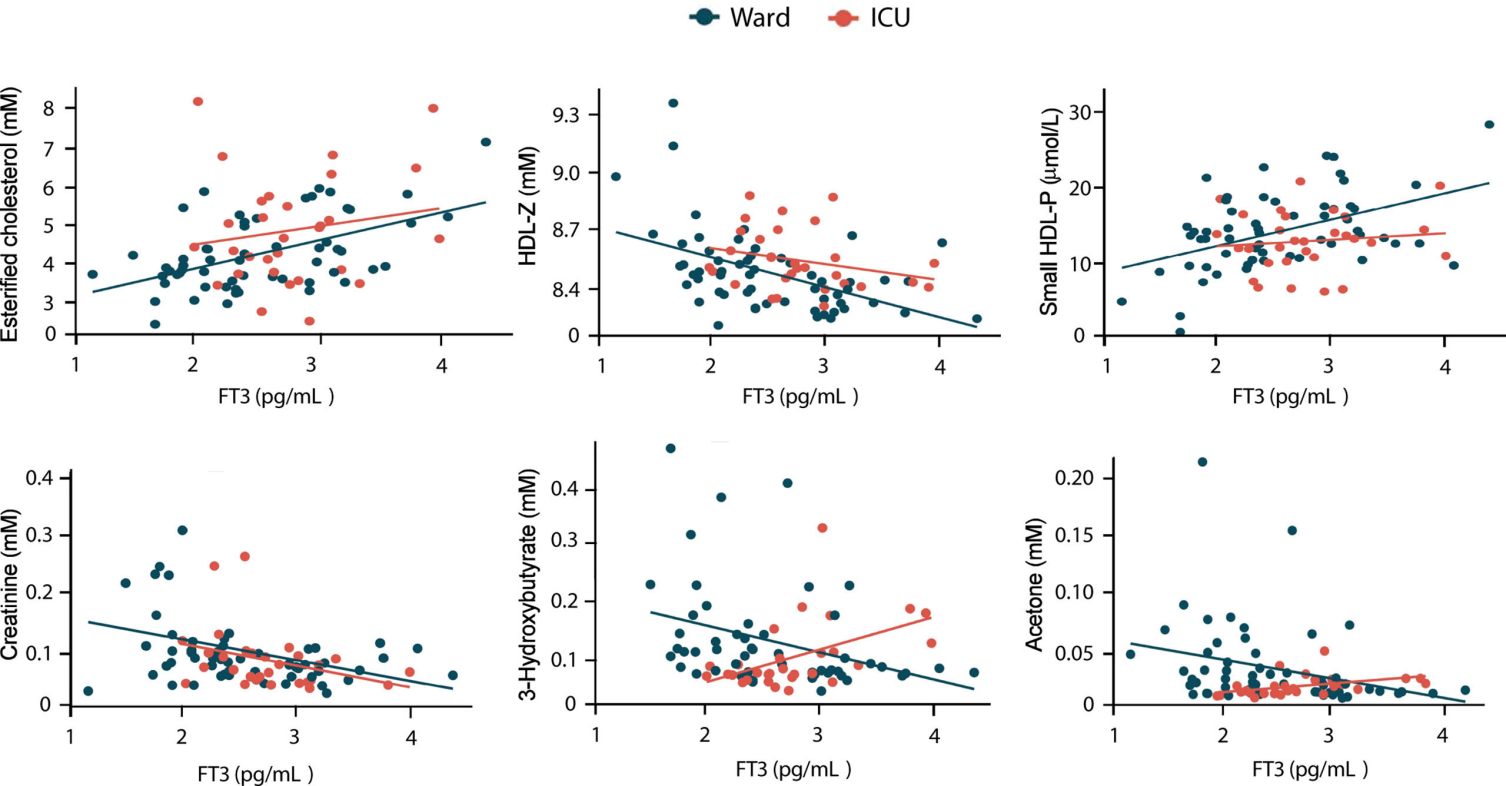
Correlation of metabolomic parameters with FT3 levels in hospitalized COVID-19 patients. Univariate linear regression analysis of the indicated metabolomic parameters in ward and ICU patient is shown.

We then compared the correlation of these 6 metabolic parameters with FT3 for all ward patients and also for all ICU patients. Acetone and 3-hydroxybutyrate were the only two metabolites that correlated differently with FT3 in ward and in ICU, negatively with FT3 in ward patients but positively in ICU patients (**Figure 4**). The interaction analysis, by testing the slopes in the ward and ICU against each other, revealed that the slopes in ward and ICU were statistically different for both 3-hydroxybutyrate (p=0.001) and acetone (p=0.019). Multiple linear regression analysis of these two metabolites with FT3 in ICU patients showed a relationship with FT3 (3-hydroxybutyrate p=0.002 and acetone p=0.007), independently of age, sex or BMI.

## Discussion

Previous studies, confirmed by our data, concluded that euthyroid and LT4-treated patients do not show significant differences in terms of COVID-19 severity and outcome (36, 37). After analysis of the different biochemical, hormonal, cytokine and metabolomic parameters between euthyroid and LT4-treated patients in the ward and in the ICU, significant differences were only found in TSH and ferritin between the two ward hospitalized groups and in TSH and FT4 between the two ICU groups. Thus, the continued administration of the usual LT4 dose allows hypothyroid patients to achieve median FT3 levels around physiological values, with no evidence for significant differences with respect to euthyroid patients during COVID-19 hospitalization. Accordingly, euthyroid and LT4-treated patients did not show evidence for significant differences in parameters such as IL-6 or some metabolites described here as FT3 dependent.

COVID-19 patients have been reported to present an euthyroid low T3 syndrome (14–21), and low FT3 has been associated to poor prognosis in COVID-19 (19, 20). In addition, age, with a higher incidence of comorbidity factors, is a poor prognosis factor in COVID-19 (2, 3). Here we show that age and FT3 levels are negatively correlated in COVID-19 patients and that parameters that predict poor prognosis are associated with low FT3, and/or with age. Therefore, age should be considered before contemplating that SARS-CoV-2 causes a low T3 syndrome. Among the different antiviral cytokines, elevated levels of IL-10 and IL-6 are predictors of COVID-19 severity (6). Both show a statistical increase in the low-FT3 group, on average 15 years older than the high-FT3 group, but only IL-6 is negatively associated to FT3 independently of age, BMI and sex, whereas IL-10 is positively related to age and independent of FT3.

It has been reported that T4 conversion to T3 in LT4 treated patients may be inefficient, especially in LT4-athyreotic patients treated with high LT4 doses, which can display a diminished FT3/FT4 ratio compared to euthyroid patients (7, 38, 39). However, our data did not show statistically significant differences in the FT3/FT4 ratio between the 4 groups of COVID-19 patients. In contrast, a significantly lower FT3/FT4 ratio was found in the low-FT3 group compared to the high-FT3 group in non-critical patients, and the low FT3/FT4 patients also show an increase of parameters that predict a worse outcome. It should be again considered that the cohorts of COVID-19 patients studied here presented a different mean age, as the patients with the lower FT3/FT4 ratio were also significantly older. This would be consistent with data previously obtained in aged patients and may reflect a decrease in T4 to T3 conversion with age, which may be part of the aging process (40).

Energy homeostasis changes dramatically during infection. The metabolic pathways sense this pathological situation producing changes in their status, as an activation of the immune system is required to mount the defense strategy. Dietary and metabolic adaptations modulated in the context of infection may increase survival of the host (41, 42). A cluster of 6 metabolic parameters, esterified cholesterol, HDL-Z, creatinine, small HDL-P, acetone and 3-hydroxybutyrate defines the metabolomic profile of non-critical hospitalized Covid-19 patients with low FT3. The higher levels of esterified cholesterol, small HDL-P HDL and lower diameter (Z) in low FT3 patients in both ward and ICU patients agrees with the fact that hypothyroidism is one the most common causes of secondary dyslipidemia (43, 44). However, acetone and 3-hydroxybutyrate, two ketogenic metabolites, showed a negative correlation with FT3 levels and also with age in non-critical hospitalized patients, but showed a positive correlation with FT3 levels not associated with age, in critical patients. The ketone bodies, acetoacetate, 3-hydroxybutyrate and acetone, a spontaneous breakdown product of acetoacetate, are products of the fatty acid metabolism serving during states of energy deficit as an alternative source of ATP (45), which is required for a proper activation of the host immune response (46). 3-Hydroxybutyrate is generated in different scenarios such as low food intake (fasting), carbohydrate restrictive diets, starvation and prolonged intense exercise (11, 47). The parental/enteral nutrition in the ICU patients should modify the source of nutrients and metabolites used to generate basal energy expenditure and to cope with the infection. 3-hydroxybutyrate is an important metabolic substrate for energy production during prolonged fasting and the generation of ketone bodies is strongly related to the amount and type of caloric intake. Caloric restriction, low FT3 and high 3-hydroxybutyrate have been proposed to be highly beneficial for adequate ageing in healthy individuals (10, 11, 47). However, in the context of an infectious process, nutrient restriction may have both pathogenic and beneficial effects. In this respect, it has been shown that different metabolic states of the host are required for an optimal response to infection, with a difference between bacterial and viral pathogens (42, 48). Here we show that in elderly COVID-19 patients with low levels of FT3, high levels of 3-hydroxybutyrate may not be a good prognostic factor.

In summary, here we show that maintaining the LT4 replacement therapy to hypothyroid patients during COVID-19 hospitalization allows them to display FT3 and FT3-dependent parameters, disease severity and outcome, with no detectable statistical differences with respect to euthyroid patients. We also evidence an inverse age-FT3 relationship in COVID-19 independent of sex and BMI. In addition, we found that non-critical hospitalized COVID-19 patients with low FT3 show higher IL-6 levels and a specific metabolomic profile with higher ketogenesis.

## Data availability statement

The raw data supporting the conclusions of this article will be made available by the authors, without undue reservation.

## Ethics statement

The studies involving human participants were reviewed and approved by the Institutional Ethics Committee of the Hospital Clínico San Carlos (HCSC) (n°=20/397-E_COVID). The patients/participants provided their written informed consent to participate in this study.

## Author contributions

IA and JM-S collected the data from COVID-19_URG-HCSC registry. EA, SE-S, MT and SA analyzed the biobank data. VG, RB, JP-P and PO confirmed and analyzed the drug administration to the patients. CA, MT, DR-M, NA and NM-M analyzed the hormone, metabolomic and cytokine. IA, LB, AA and SA wrote the manuscript. NA, AA and SA were involved in acquisition of funding. All authors contributed to the article and approved the submitted version.

## Funding

ID2020-116146RB-I00 from the Ministerio de Ciencia e Innovación with European Regional Development Funds (FEDER), BMD-3724 from the Comunidad de Madrid, 202020E169 from the CSIC, 2020PANDE00082 from the Generalitad de Cataluña and Fundación Hay Esperanza.

## Acknowledgments

We thank to Elena Molina (Biobank), Henar Gonzalez (Unidad de Innovación) and to Irene Garcia (Investigación) for their involvement in this manuscript and data and to the different professionals carefully dedicated to the patients included in this study. We acknowledge support of the publication fee by the CSIC Open Access Publication Support Initiative through its Unit of Information Resources for Research (URICI).

## Conflict of interest

The authors declare that the research was conducted in the absence of any commercial or financial relationships that could be construed as a potential conflict of interest.

## Publisher’s note

All claims expressed in this article are solely those of the authors and do not necessarily represent those of their affiliated organizations, or those of the publisher, the editors and the reviewers. Any product that may be evaluated in this article, or claim that may be made by its manufacturer, is not guaranteed or endorsed by the publisher.
