# Supplementary material for: Free triiodothyronine levels and age influences the metabolic profile and COVID-19 severity parameters in euthyroid and levothyroxine-treated patients
Source: Front Endocrinol (Lausanne). 2022 Nov 9;13:1025032. doi: 10.3389/fendo.2022.1025032 (PMC9682171; doi:10.3389/fendo.2022.1025032)
Supplement: Supplementary file 1 [file DataSheet_1.pdf]

Supplementary Material

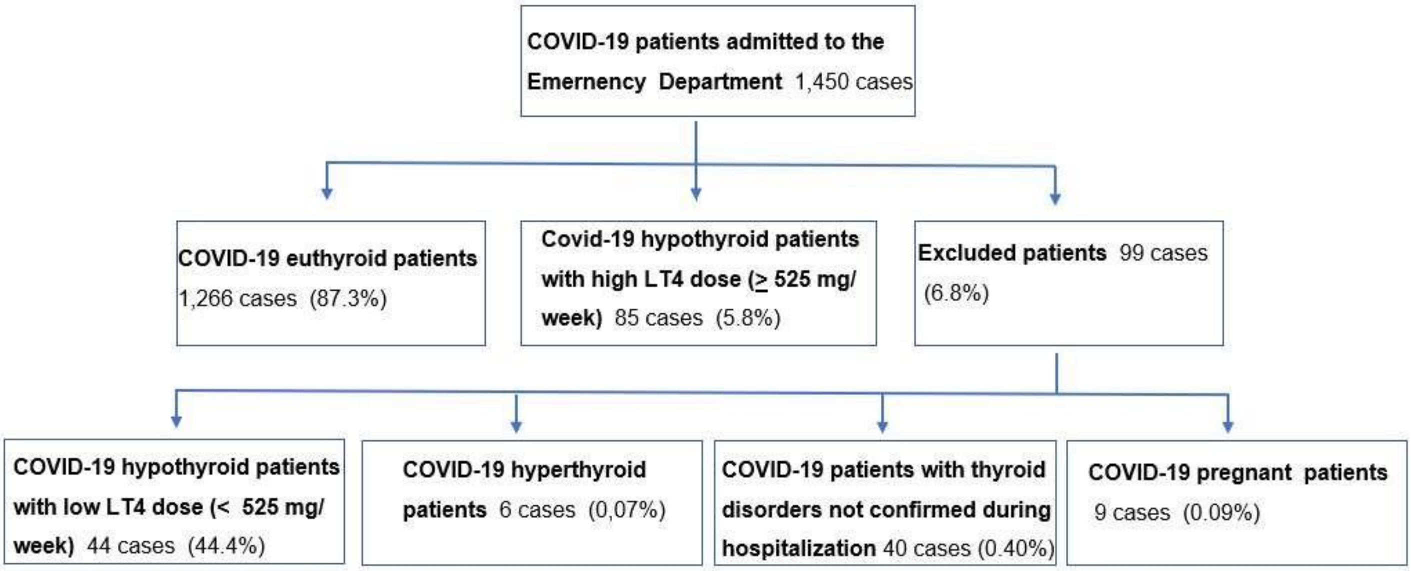

Supplementary figure 1. Flowchart of patient inclusion

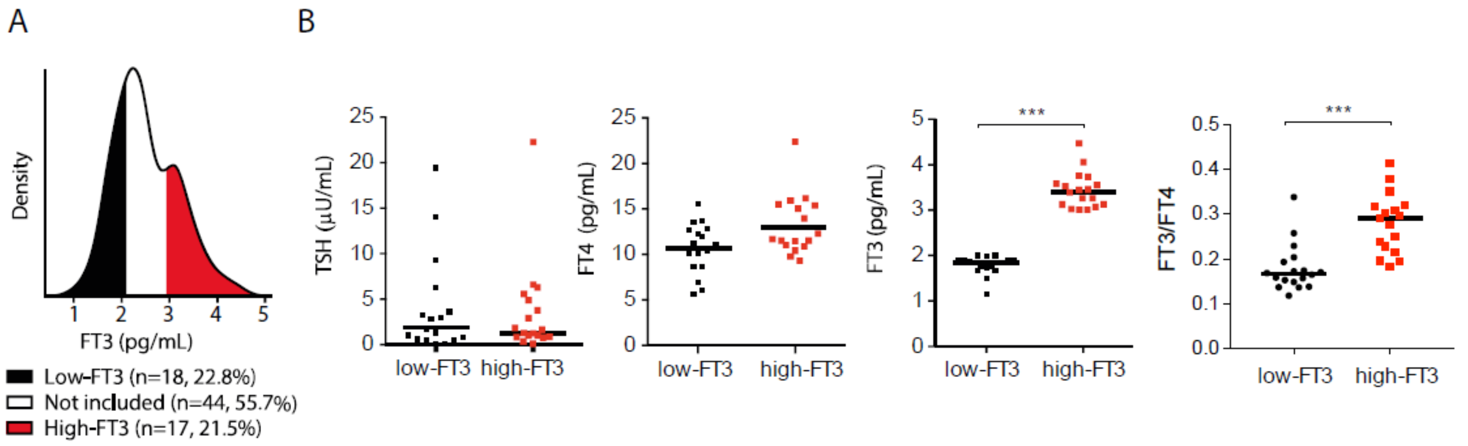

Supplementary Figure 2. Distribution of ward hospitalized COVID-19 patients according to their FT3 levels. A) Patients distribution. **B)** TSH and thyroid hormone levels and FT3/FT4 ratio in low-FT3 and high-FT3 patients. Comparisons were made with two-tailed Mann-Whitney test. ***$p<0.0001$.

Supplementary Table 1. Coefficient of variation of the different biochemical parameters.

Parameters	Control	Media	CV %
Leukocytes x 10^6/uL	level 1	3.4	1.3
	level 2	9.2	1.31
	level 3	20.8	1.3
Hemoglobin g/dL	level 1	4.9	0.7
	level 2	15.5	0.77
	level 3	12.3	0.56
Lymphocytes x 10^3/uL	level 1	1.6	1.86
	level 2	2.7	2.1
	level 3	3.1	3.61
Neutrophils x $10^{3/}$uL	level 1	1.5	2.3
	level 2	5.2	1.62
	level 3	13.7	1.68
Platelets x 10^3/uL	level 1	72	2.02
	level 2	228	1.81
	level 3	432	1.56
Alanine aminotransferase U/L	level 1	27.89	3.56
	level 2	85.35	2.57
	level 3	191.24	2.41
Aspartate aminotransferase U/L	level 1	43.8	3.01
	level 2	119.5	2.59
	level 3	268	2.64
C-reactive protein mg/dL	level 1	0.9	3.7
	level 2	2.9	3.3
	level 3	4.7	1.9
D-Dimer ng/mL	level 1	667	5
	level 2	2047	5
	level 3		
Fibrinogen mg/dL	level 1	342	2
	level 2		
	level 3		
Ferritin ng/mL	level 1	24	2.7
	level 2	138	2.9
	level 3	304.4	3.3
Lactate dehydrogenase U/L	level 1	258.6	2.83
	level 2	358.6	2.23
	level 3	933.1	1.5

CV: Coefficient of variation. The quality of the methods is assessed through the External Quality Assurance Program of the Spanish Society of Clinical Chemistry, which is carried out on a monthly basis.

Supplementary Table 2. Drugs administered to hospitalized COVID-19 patients on the day before TSH, FT3 and FT4 blood sampling.

	Ward<br>Euthyroid (n=40)	Ward<br>LT4-treated (n=39)	ICU<br>Euthyroid (n=29)	ICU<br>LT4-treated (n=9)	p value
Anticoagulants	87.0%	91.0%	75.0%	61.0%	0.195
Antidepressants	4.9%	2.7%	8.0%	7.8%	>0.9999
Diuretics	7.3%	8.1%	13.0%	7.9%	0.803
Antiepileptics	4.9%	5.4%	8.0%	0.0%	0.900
Antipsychotic	4.9%	10.0%	13.0%	15.0%	0.486
Beta blockers	14.6%	27.0%	5.4%	7.8%	0.202
Corticosteroids	51.5%	51.3%	62.0%	53.8%	>0.9999
NAIDS	11.0%	0.0%	0.0%	0.0%	>0.9999
Levothyroxine	0.0%	100.0%	0.0%	100.0%	<0.0001

Non-categorical data are entered as percentages and analyzed by X^2. *Anticoagulants:* Bemiparin Enoxaparin Heparin. Tinzaparin; *antidepressants:* Mirtazapine; *antidiuretics:* Allopurinol. Furosemide; *antiepileptics:* Pregabalin. Topiramate; Lacosamide; antipsychotic. Quetiapine. Risperidone. Sulpiride; *beta blockers:* Atenolol. Bisoprolol. Carvedilol. Metoprolol; *corticosteroids:* Betamethasone. Dexamethasone. Hydrocortisone. Methylprednisolone. Prednisone; *Non-steroidal anti-inflammatory drugs*: dexketoprofen.

Supplementary Table 3. Circulating TSH, FT4, FT3 and FT3/FT4 in COVID-19 hospitalized patients.

	Ward Euthyroid (n=40)	Ward LT4-treated (n=39)	ICU Euthyroid (n=29)	ICU LT4-treated (n=9)	p value
TSH (μU/ml)					
Median [Q1:Q3]	1.04 [0.82;2.1]	3.31[0.64;6.77]**	1.13[0.49-2.34]	2.81[2.27;3.26] #	0.004
Out above	2.5%	37.5%	3.4%	3.26%	
Out below	12.5%	22.5%	20.7%	0.0%	
FT4 (pg/ml)					
Median [Q1:Q3]	11.43[10.15;13.74]	10.64[8.97;12.29]	12.32[10.72;13.79]	9.84[8.11;11.06] #	0.016
Out above	5.0%	0.0%	6.9%	11.1%	
Out below	0.0%	0.0%	0.0%	0.0%	
FT3 (pg/ml)					
Median [Q1:Q3]	2.77[2.15;3.23]	2.41[2.07;2.93]	2.67[2.47;3.08]	2.78[2.47;3.08]	0.115
Out above	7.5%	5.0%	6.8%	0.0%	
Out below	35.0%	52.5%	24.1%	33.3%	
FT3/FT4					
Median [Q1:Q3]	0.23[0.18-0.29]	0.25[0.17-0.29]	0.23[0.19-0.25]	0.29[0.22-0.36]	0.403
Out above	22.5%	25.6%	24.1%	22.2%	
Out below	22.5%	15.4%	24.1%	22.2%	

Medians and interquartile range [Q1:Q3] of TSH, FT4, FT3 and FT3/FT4 from patients described in Table 1, are shown. Comparisonswere made with Kruskal-Wallis test followed by false discovery rate by two-stage linear step-up procedure of Benjamini, Krieger and Yekutieli. In the table p values correspond to ANOVA analysis. Multiple comparisons were also performed: Only statistical differences between both ward or both ICU groups and between the two euthyroid or LT4-treated groups were considered. **P<0.01, between euthyroid ward and LT4-treated ward patients. # p<0.05 between euthyroid ICU and LT4-treated ICU patients. The percentage of patients with hormone values outside of the physiological range is also indicated.

Supplementary Table 4. Low molecular weight metabolites, lipid, lipoprotein and glycoprotein profiles determined by ^{1}H-NMR in serum from hospitalized COVID-19 patients.

	Ward<br>Euthyroid (n=40)	Ward<br>LT4-treated (n=39)	ICU<br>Euthyroid (n29)	ICU<br>LT4-treated (n=9)	p value
Low molecular weight metabolites					
3-Hydroxybutyrate (mM)	0.10 [0.05;0.16]	0.07 [0.05;0.10]	0.06 [0.05;0.10]	0.06 [0.03;0.09]	0.436
Acetate (mM)	0.03 [0.03;0.04]	0.03 [0.02;0.03]	0.04 [0.03;0.05]	0.03 [0.02;0.04]	0.028
Acetone (mM)	0.02 [0.01;0.04]	0.02 [0.01;0.04]	0.02 [0.01;0.02]	0.01 [0.01;0.02]	0.153
Alanine (mM)	0.33 [0.29;0.40]	0.33 [0.30;0.43]	0.34 [0.27;0.40]	0.36 [0.31;0.42]	0.701
Creatinine (mM)	0.07 [0.05;0.10]	0.07 [0.05;0.09]	0.06 [0.04;0.09]	0.08 [0.03;0.09]	0.739
Creatine (mM)	0.04 [0.01;0.08]	0.04 [0.02;0.06]	0.06 [0.04;0.09]	0.09 [0.05;0.12]	0.045
Formate (mM)	0.01 [0.01;0.01]	0.01 [0.01;0.01]	0.01 [0.01;0.01]	0.01 [0.01;0.02]	0.998
Glucose (mM)	3.75 [3.35;4.55]	4.16 [3.71;4.95]	5.73 [4.34;7.20] ***	5.48 [4.51;7.42]	<0.0001
Glutamate (mM)	0.15 [0.12;0.20]	0.15 [0.12;0.19]	0.18 [0.14;0.21]	0.17 [0.16;0.17]	0.112
Glutamine (mM)	0.39 [0.34;0.44]	0.34 [0.30;0.43]	0.33 [0.23;0.44]	0.32 [0.29;0.43]	0.241
Glycine (mM)	0.28 [0.24;0.34]	0.27 [0.24;0.30]	0.33 [0.26;0.40]	0.33 [0.26;0.36]	0.034
Histidine (mM)	0.07 [0.07;0.08]	0.07 [0.06;0.08]	0.07 [0.06;0.08]	0.06 [0.05;0.08]	0.429
Lactate (mM)	1.48 [1.07;1.92]	1.26 [0.93;1.89]	1.34 [0.98;1.82]	1.88 [1.58;2.30]	0.093
Threonine (mM)	0.24 [0.23;0.26]	0.22 [0.20;0.26]	0.31 [0.25;0.38]**	0.27 [0.22;0.31]	<0.0001
Tyrosine (mM)	0.06 [0.05;0.06]	0.05 [0.04;0.06]	0.06 [0.04;0.07]	0.07 [0.06;0.07]	0.046
Valine (mM)	0.23 [0.21;0.27]	0.23 [0.19;0.26]	0.29 [0.24;0.33]**	0.28 [0.23;0.32]*	<0.001
Isoleucine (mM)	0.04 [0.04;0.05]	0.04 [0.03;0.05]	0.04 [0.03;0.06]	0.05 [0.04;0.06]	0.904
Leucine (mM)	0.12 [0.11;0.14]	0.11 [0.10;0.13]	0.15 [0.12;0.19]*	0.14 [0.13;0.16]	<0.0001
Lipid metabolites					
Esterified cholesterol (mM)	3.90 [3.40;4.91]	3.87 [3.55;4.49]	4.20 [3.61;4.81]	4.27 [3.70;4.75]	0.582
Free cholesterol (mM)	2.56 [2.09;2.91]	2.52 [2.05;2.88]	2.99 [2.54;3.58]*	3.04 [2.31;5.06]	0.005
Triglycerides (mM)	1.79 [1.33;2.57]	2.01 [1.38;2.58]	2.32 [1.86;2.98]	2.32 [1.63;2.71]	0.132
Glycerophospholipids (mM)	3.30 [3.10;3.69]	3.11 [2.84;3.72]	3.62 [3.22;3.94]	3.79 [3.16;4.82]	0.121
Phosphatidylcholine (mM)	2.86 [2.71;3.32]	2.80 [2.50;3.29]	3.19 [2.86;3.51]	3.26 [2.79;4.37]	0.14
Sphingomyelin (mM)	0.85 [0.76;0.99]	0.87 [0.77;0.97]	0.85 [0.71;0.97]	0.94 [0.81;0.98]	0.751
Lysophosphatidylcholine (mM)	0.68 [0.53;0.93]	0.71 [0.63;0.81]	0.63 [0.52;0.74]	0.60 [0.51;0.71]	0.157
PUFA (mM)	1.78 [1.47;2.39]	1.98 [1.49;2.51]	1.94 [1.52;2.83]	2.24 [2.08;2.61]	0.557

PUFA (mM)	4.84 [3.60;5.79]	4.81 [4.12;6.00]	5.31 [4.27;6.37]	4.95 [4.08;5.95]	0.525
PUFA (mM)	3.35 [2.62;4.14]	3.58 [2.54;4.12]	3.06 [2.23;3.79]	2.69 [2.52;3.30]	0.228
PUFA (mM)	2.61 [2.01;3.43]	2.68 [2.28;3.19]	2.70 [2.13;3.34]	3.04 [2.43;4.15]	0.589
Linoleic (mM)	3.95 [3.01;4.44]	3.68 [3.25;4.54]	4.85 [3.93;5.70]**	5.60 [3.89;6.70] #	0.002
SFA (mM)	4.88 [4.10;5.56]	5.09 [4.38;5.77]	5.35 [4.74;6.41]	6.20 [4.58;7.91]	0.085
ω6 & ω7 fatty acids (mM)	4.50 [3.89;5.14]	4.52 [4.07;5.24]	5.48 [4.64;6.24]	6.17 [4.58;7.12]	0.053
ω9 fatty acids (mM)	3.79 [3.03;4.45]	3.77 [3.07;4.54]	4.11 [3.66;4.92]	4.54 [3.53;5.05]	0.115
ω3 fatty acids (mM)	0.34 [0.28;0.49]	0.38 [0.28;0.48]	0.42 [0.30;0.52]	0.54 [0.42;0.75]	0.136
DHA (mM)	0.08 [0.06;0.11]	0.09 [0.07;0.11]	0.10 [0.06;0.14]	0.08 [0.06;0.15]	0.576
AA + EPA (mM)	0.65 [0.54;0.76]	0.60 [0.52;0.78]	0.62 [0.52;0.80]	0.57 [0.50;0.67]	0.661
Lipoprotein profile					
VLDL-Cholesterol (mg/dL)	23.2 [16.8;31.1]	25.9 [17.6;31.9]	28.6 [19.8;38.5]	27.9 [19.3;37.1]	0.36
IDL-Cholesterol (mg/dL)	18.1 [12.5;21.7]	18.4 [11.5;21.5]	22.3 [19.2;26.8]**	24.0 [19.6;28.2] #	0.001
LDL-Cholesterol (mg/dL)	104 [88.5;124]	110 [88.7;123]	118 [96.3;139]	118 [95.4;135]	0.188
HDL-Cholesterol (mg/dL)	44.8 [38.1;54.3]	42.8 [36.9;50.1]	41.1 [32.0;46.9]	40.8 [35.9;59.2]	0.329
VLDL-Triglycerides (mg/dL)	85.7 [62.4;121]	88.8 [67.7;122]	107 [78.1;148]	104 [66.0;143]	0.251
IDL-Triglycerides (mg/dL)	16.7 [12.9;19.9]	16.6 [12.1;19.5]	20.8 [17.0;23.0]**	20.5 [18.1;25.1] #	0.001
LDL-Triglycerides (mg/dL)	21.0 [16.9;25.7]	21.9 [16.3;26.7]	27.9 [23.6;31.5]**	31.3 [23.8;39.3] #	0.001
HDL-Triglycerides (mg/dL)	21.7 [18.2;25.5]	21.1 [18.6;24.8]	22.7 [18.7;27.9]	22.9 [21.9;26.7]	0.655
VLDL-P (nM)	62.9 [46.2;87.5]	63.6 [48.9;90.9]	78.2 [55.7;106]	75.3 [47.8;106]	0.297
Large VLDL-P (nM)	1.81 [1.31;2.06]	1.70 [1.47;2.28]	1.77 [1.45;2.38]	1.86 [1.52;2.31]	0.853
Medium VLDL-P (nM)	5.86 [4.68;7.46]	6.79 [4.88;8.31]	7.09 [5.58;10.1]	7.22 [6.28;8.86]	0.122
Small VLDL-P (nM)	55.8 [40.0;74.7]	55.4 [41.3;81.2]	69.0 [48.1;91.3]	66.2 [41.9;94.5]	0.339
LDL-P (nM)	1,101 [899;1,349]	1,091 [997;1,258]	1,298 [1,081;1,525]	1,291 [1,083;1,460]	0.05
Large LDL-P (nM)	163 [140;183]	180 [148;191]	191 [164;223]	182 [163;212]	0.054
Medium LDL-P (nM)	287 [234;370]	336 [270;383]	384 [298;516]	424 [313;480]	0.017
Small LDL-P (nM)	658 [526;711]	594 [546;682]	660 [569;781]	680 [601;830]	0.274
HDL-P (μmol/L)	25.5 [21.3;28.5]	24.7 [22.1;27.7]	23.9 [20.0;26.4]	24.1 [22.1;32.5]	0.251
Large HDL-P (μmol/L)	0.30 [0.28;0.33]	0.30 [0.28;0.33]	0.32 [0.29;0.37]	0.34 [0.28;0.42]	0.097
Medium HDL-P (μmol/L)	11.5 [10.7;12.6]	11.1 [9.96;12.3]	10.9 [10.4;12.8]	11.8 [10.4;14.4]	0.657
Small HDL-P(μmol/L)	13.6 [9.93;16.6]	13.3 [12.0;16.8]	11.3 [8.46;13.5]*	12.5 [12.2;16.8]	0.041
VLDL-Z (nm)	42.1 [42.0;42.2]	42.2 [42.1;42.3]	42.1 [42.0;42.3]	42.1 [42.1;42.2]	0.417
LDL-Z (nm)	21.1 [21.0;21.4]	21.3 [21.1;21.3]	21.3 [21.1;21.5]	21.3 [21.1;21.6]	0.185

HDL-Z (nm)	8.48 [8.36;8.55]	8.42 [8.34;8.51]	8.55 [8.46;8.65]	8.48 [8.41;8.52]	0.2053
Non-HDL-P (nM)	1,131 [919;1,362]	1137 [1034;1284]	1,330 [1,147;1,584]	1.365 [1,151;1,552]	0.02
Total-P/HDL-P	48.6 [38.8;57.1]	50.0 [39.3;56.8]	57.5 [49.5;66.9]**	59.4 [49.2;63.7]	0.003
LDL-P/HDL-P	45.4 [36.8;52.7]	47.2 [37.0;54.3]	54.2 [46.6;62.1]***	56.8 [46.0;61.5]	0.003
VLDL-TG/VLDL-C	3.82 [3.55;4.05]	3.63 [3.40;4.14]	3.77 [3.46;4.11]	4.00 [3.50;4.36]	0.693
IDL-TG/IDL-C	0.98 [0.90;1.04]	0.96 [0.90;1.04]	0.89 [0.84;0.97]	0.89 [0.83;0.95]	0.018
LDL-TG/LDL-C	0.20 [0.16;0.25]	0.19 [0.15;0.23]	0.24 [0.19;0.28]	0.20 [0.18;0.30]	0.114
HDL-TG/HDL-C	0.51 [0.37;0.65]	0.50 [0.36;0.66]	0.59 [0.43;0.79]	0.59 [0.43;0.68]	0.35
Total Cholesterol (mg/dl)	191 [170;214]	193 [175;211]	215 [182;235]	227 [185;242]	0.073
Total Triglycerides (mg/dL)	140 [110;188]	149 [116;186]	175 [147;226]	173 [144;218]	0.067
Glycoprotein profile					
Glyc-B (µmol/L)	476 [434;526]	479 [441;514]	512 [451;555]	517 [498;574]	0.056
Glyc-F (µmol/L)	258 [233;273]	268 [244;294]	272 [249;306]	277 [262;301]	0.133
Glyc-A (µmol/L)	894 [791;1,031],	921 [813;1,060]	1,053 [918;1,204]**	1101 [994;1243]#	0.001
GlycBound.Glyc.Free	5.99 [5.46;6.61]	6.03 [5.58;6.47]	6.44 [5.67;6.99]	6.50 [6.27;7.22]	0.053
H/W Glyc-B	26.4 [23.6;30.0]	26.3 [23.7;30.2]	30.6 [26.3;34.5]	30.8 [28.6;35.6]	0.001
H/W Glyc-A	5.21 [4.76;5.87]	4.90 [4.70;5.43]	5.39 [5.18;6.16]**	5.61 [5.44;5.81]#	0.001

Medians and interquartile range [Q1-Q3] of the indicated cytokines are shown. Comparisons were made with Kruskal-Wallis test followed by false discovery rate by two-stage linear step-up procedure of Benjamini, Krieger and Yekutieli. . In the table p values correspond to ANOVA analysis. Multiple comparisons were also performed. Only statistical differences between both ward or both ICU groups and between the two euthyroid or LT4-treated groups were considered. No statistical differences between euthyroid ward and LT4-treated ward patients and between and euthyroid ICU and LT4-treated ICU patients were found. *p<0.05, **p<0.01, ***<0.001 statistical differences between euthyroid ward and euthyroid ICU patients; # p <0.05 statistical differences between LT4-treated ward and LT4-treated patients ICU. AA: Arachidonic fatty acid; DHA: Docosahexaenoic; EPA: Eicosapentaenoic acid; Gly: Glycoprotein; H: high; HDL: High density lipoprotein; IDL: HDL: High density lipoprotein; IDL: Intermediate density Lipoprotein; P: Particles; PUFA: Polyunsaturated fatty acids (four different PUFA signals); SFA: Saturated fatty acids; TG: Triglycerides; VLDL: Very low-density lipoprotein; w: Omega; W: width; Z: Diameter.

Supplementary Table 5. Baseline characteristics, laboratory data and outcome in ward hospitalized COVID-19 patients.

	Low-FT3/FT4 (n=18)	High-FT3/F4 (n=17)	p-value
Gender and age			
Male (n=7, 4)	38.9%	23.5%	0.471
Female (n=11,13)	61.1%	76.5%	0.471
Age, years (n=18,17)	78.1±9	58,0±13	<0.001
Laboratory data at the day of TSH, FT4 and FT3			
TSH (n=18,17)	1.51 [0.42;3.12]	0.99 [0.85;5.94]	0.683
FT4 (n=18,17)	12.7 [11.1;14.2]	9.2 [7.5;10.4]	<0.001
FT3 (n=18,17)	1.90 [1.75;2.1]	3.22 [2.83;3.64]	<0.001
Neutrophils /Lymphocytes ratio	6.9 [5.6;20.2]	2.2 [1.5;3.8]	<0.001
ALT U/L (n=18,17)	22.3 [18.1;50.0]	24.1 [14.3;59.2]	0.831
AST U/L (n=18,17)	31.5 [17.3;60.8]	23.0 [20.0;29.0]	0.395
C-reactive protein mg/dL (n=18,17)	12.7 [1.82;13.90]	2.91 [0.58;6.38]	0.047
D-Dimer ng/dL (n=18,17)	3,867 [723;6,126]	713 [445;1,287]	0.015
Fibrinogen mg/dL (n=18,17)	583 [413;714]	602 [396;670]	0.752
Ferritin ng/dL (n=18,17)	514 [232;1045]	215 [72;664]	0.123
LDH U/L (n=18,17)	517 [448;948]	519 [454;579]	0.470
IL-6 (n=18,17)	314 [2.1;2,057]	2.5 [1.8;228]	0.049
IL-10 (n=18,17)	5.0 [4.2;9.0]	4.2 [3.6;5.2]	0.054
Outcome			
Exitus (n=5,1)	27.8%	5.8%	0.177

Sixty-six per cent of the low-F3/FT4 patients are the same as those in the low-F3 group and 41.2% of the high-F3/FT4 patients are the same as those in the high-F3 group. Non-categorical data are entered as percentages and were analyzed by Fisher exact test. Parametrical data are shown as means±S.D and were analyzed by two-tailed Student´s *t* test. Non-parametric data are expressed as medians and interquartile range [Q1;Q3] and comparisons were made with Mann-Withney test. AST: Aspartate aminotransferase; ALT: Alanine aminotransferase, LDH: Lactate dehydrogenase.

Supplementary Table 6. Low molecular weight metabolite, lipid, lipoprotein and glycoprotein profiles determined by ^{1}H-NMR in serum from ward hospitalized COVID-19 patients.

	Low-FT3 (n=18)	High-FT3 (n=17)	p-value
Low molecular weight metabolites			
3-Hydroxybutyrate (mM)	0.13 [0.09;0.24]	0.05 [0.05;0.07]	0.004
Acetate (mM)	0.03 [0.03;0.04]	0.03 [0.02;0.04]	0.552
Acetone (mM)	0.03 [0.02;0.07]	0.01 [0.01;0.02]	0.008
Alanine (mM)	0.32 [0.28;0.41]	0.32 [0.28;0.43]	0.552
Creatinine (mM)	0.09 [0.06;0.22]	0.06 [0.04;0.08]	0.044
Creatine (mM)	0.02 [0.00;0.06]	0.03 [0.01;0.08]	0.921
Formate (mM)	0.01 [0.01;0.02]	0.01 [0.01;0.01]	0.771
Glucose (mM)	3.77 [3.08;4.70]	3.79 [3.36;4.32]	0.947
Glutamate (mM)	0.14 [0.12;0.20]	0.16 [0.11;0.19]	0.909
Glutamine (mM)	0.37 [0.31;0.45]	0.36 [0.32;0.39]	0.597
Glycine (mM)	0.28 [0.24;0.34]	0.30 [0.27;0.35]	0.291
Histidine (mM)	0.07 [0.05;0.08]	0.07 [0.07;0.08]	0.509
Lactate (mM)	1.47 [1.17;1.96]	1.39 [1.15;1.90]	0.654
Threonine (mM)	0.22 [0.20;0.24]	0.23 [0.20;0.25]	0.664
Tyrosine (mM)	0.05 [0.04;0.06]	0.05 [0.04;0.06]	0.895
Valine (mM)	0.22 [0.19;0.25]	0.24 [0.21;0.26]	0.210
Isoleucine (mM)	0.04 [0.03;0.05]	0.04 [0.04;0.06]	0.222
Leucine (mM)	0.12 [0.10;0.13]	0.12 [0.11;0.14]	0.959
Lipid profile			
Esterified cholesterol (mM)	3.62 [3.52;3.75]	4.32 [3.74;5.14]	0.007
Free cholesterol (mM)	2.72 [2.55;3.09]	2.56 [2.18;2.89]	0.373
Triglycerides (mM)	1.78 [1.40;2.39]	2.21 [1.58;2.68]	0.198
Glycerophospholipids (mM)	3.17 [2.87;3.65]	3.18 [3.00;3.97]	0.235
Phosphatidylcholine (mM)	2.74 [2.61;3.12]	2.82 [2.71;3.59]	0.137
Sphingomyelin (mM)	0.91 [0.77;0.98]	0.88 [0.77;1.00]	0.921
Lysophosphatidylcholine (mM)	0.64 [0.45;0.70]	0.73 [0.63;0.98]	0.032
PUFA (mM)	1.67 [1.48;2.11]	1.94 [1.25;2.95]	0.338
PUFA (mM)	4.59 [3.44;5.15]	4.50 [3.94;5.79]	0.262
PUFA (mM)	3.31 [2.39;4.01]	3.04 [2.56;3.88]	0.869
PUFA (mM)	2.35 [2.24;3.32]	3.01 [2.30;3.44]	0.210
Linoleic (mM)	3.65 [2.95;4.44]	4.26 [3.45;5.00]	0.106
SFA (mM)	5.18 [4.17;5.73]	4.93 [4.54;5.76]	0.974
6 & 7 fatty acids (mM)	4.38 [3.58;4.80]	4.66 [4.32;5.89]	0.106
9 fatty acids (mM)	3.57 [3.13;4.03]	3.92 [3.42;4.81]	0.129
3 fatty acids (mM)	0.38 [0.28;0.46]	0.43 [0.33;0.54]	0.373
DHA(mM)	0.09 [0.06;0.11]	0.08 [0.05;0.10]	0.552
AA + EPA (mM)	0.66 [0.54;0.74]	0.57 [0.48;0.63]	0.276
Lipoprotein profile			
VLDL-Cholesterol (mg/dL)	25.2 [18.6;29.5]	25.3 [22.2;33.2]	0.248
IDL-Cholesterol (mg/dL)	19.1 [16.7;21.3]	18.9 [12.6;23.5]	0.921

LDL-Cholesterol (mg/dL)	105 [89.7;114]	110 [97.9;133]	0.409
HDL-Cholesterol (mg/dL)	42.2 [35.6;45.9]	44.1 [35.6;53.8]	0.291
VLDL-Triglycerides (mg/dL)	81.2 [64.9;113]	104 [84.8;126]	0.086
IDL-Triglycerides (mg/dL)	17.1 [15.6;19.9]	18.9 [14.4;20.3]	0.552
LDL-Triglycerides (mg/dL)	22.9 [21.4;26.7]	22.3 [17.3;26.2]	0.575
HDL-Triglycerides (mg/dL)	22.4 [19.9;23.9]	22.7 [20.9;25.2]	0.552
VLDL-P (nM)	61.9 [47.4;78.5]	75.0 [58.3;94.0]	0.121
Large VLDL-P (nM)	1.70 [1.27;1.90]	1.94 [1.61;2.29]	0.069
Medium VLDL-P (nM)	6.28 [5.26;8.07]	6.35 [5.86;8.31]	0.468
Small VLDL-P (nM)	54.2 [40.3;67.2]	66.8 [51.1;83.5]	0.129
LDL-P (nM)	1,104 [1,014;1,178]	1,113 [1,061;1,409]	0.552
Large LDL-P (nM)	172 [152;183]	169 [148;201]	0.668
Medium LDL-P (nM)	326 [300;352]	325 [244;397]	0.766
Small LDL-P (nM)	589 [541;657]	678 [597;832]	0.092
HDL-P (μmol/L)	22.9 [19.9;26.5]	26.1 [22.2;31.0]	0.069
Large HDL-P (μmol/L)	0.31 [0.30;0.33]	0.29 [0.28;0.34]	0.373
Medium HDL-P (μmol/L)	11.9 [11.2;13.0]	10.8 [10.0;11.5]	0.060
Small HDL-P(μmol/L)	11.6 [7.96;13.6]	15.2 [12.0;19.8]	0.023
VLDL-Z (nm)	42.1 [42.0;42.3]	42.1 [42.0;42.2]	0.766
LDL-Z (nm)	21.2 [21.1;21.4]	21.0 [20.9;21.3]	0.075
HDL-Z (nm)	8.52 [8.45;8.65]	8.35 [8.29;8.44]	0.005
Non-HDL-P (nM)	1,137 [1089;1,199]	1,149 [1,101;1,448]	0.531
Total-P/HDL-P	53.0 [46.5;62.8]	48.8 [40.9;57.5]	0.276
LDL-P/HDL-P	50.6 [44.6;60.3]	45.2 [38.7;55.5]	0.248
VLDL-TG/VLDL-C	3.58 [3.49;3.83]	3.75 [3.28;4.01]	0.921
IDL-TG/IDL-C	0.90 [0.87;0.98]	0.96 [0.89;1.03]	0.276
LDL-TG/LDL-C	0.21 [0.19;0.25]	0.18 [0.15;0.23]	0.176
HDL-TG/HDL-C	0.52 [0.44;0.67]	0.55 [0.38;0.61]	0.621
Total Cholesterol (mg/dl)	191 [175;198]	203 [188;239]	0.166
Total Triglycerides (mg/dL)	146 [127;177]	170 [133;198]	0.248
Glycoprotein profile			
Glyc-B (μmol/L)	476 [449;517]	487 [443;522]	1.000
Glyc-F (μmol/L)	261 [230;273]	271 [254;305]	0.262
Glyc-A (μmol/L)	901 [811;995]	1002 [921;1060]	0.187
GlycBound.Glyc.Free	5.99 [5.65;6.50]	6.16 [5.59;6.56]	0.974
H/W Glyc-B	26.4 [25.1;29.1]	28.0 [26.1;30.4]	0.621
H/W Glyc-A	5.37 [5.00;5.57]	4.80 [4.57;5.88]	0.409

Medians and interquartile range [Q1;Q3] are shown. Comparisons were made with Mann-Withney test. AA: Arachidonic fatty acid; DHA: Docosahexaenoic; EPA: Eicosapentaenoic fatty acid; Gly: Glycoprotein; H: High; HDL: High density lipoprotein; IDL: Intermediate density Lipoprotein; P: Particles; PUFA: Polyunsaturated fatty acids (four different PUFA signals); SFA: Saturated fatty acids; TG: Triglycerides; VLDL: Very low-density lipoprotein; ω: Omega; W: Width; Z: Diamete
